# Effectiveness and Costs of Participant Recruitment Strategies to a Web-Based Population Cohort: Observational Study

**DOI:** 10.2196/75116

**Published:** 2025-10-06

**Authors:** Hannah Milbourn, Archie Campbell, Fiona Clark, Elly Darrah, Robin Flaig, Liz Kirby, Daniel L McCartney, Isla Mitchell, Sarah Robertson, Anne Richmond, Rosie Tatham, Zhuoni Xiao, Kerim McAteer, Caroline Hayward, Riccardo E Marioni, Andrew M McIntosh, David J Porteous, Heather C Whalley, Cathie L M Sudlow

**Affiliations:** 1 Centre for Genomic and Experimental Medicine, Institute of Genetics and Cancer, The University of Edinburgh, Western General Hospital, Edinburgh, United Kingdom; 2 Centre for Medical Informatics, Usher Institute, The University of Edinburgh, 5-7 Little France Road, Edinburgh BioQuarter—Gate 3, Edinburgh, EH16 4UX, United Kingdom, +44 (0)131 651 7869; 3 Centre for Clinical Brain Sciences, Division of Psychiatry, The University of Edinburgh, Edinburgh, United Kingdom; 4 Spirit Media Scotland, Edinburgh, United Kingdom

**Keywords:** recruitment methods, Facebook, social media, cost-effectiveness, advertisement, web-based study, cohort studies

## Abstract

**Background:**

Recruitment to population-based health studies remains challenging, with difficulties meeting target participant numbers, biosample returns, and achieving a representative sample. Few studies provide evaluations of traditional and web-based recruitment methods particularly for studies with broad inclusion criteria and extended recruitment periods. Generation Scotland (GS) is a family-based cohort study that initiated a new wave of recruitment in 2022 using web-based data collection and remote saliva sampling (for genotyping). Here, we provide an overview of recruitment strategies used by GS over the first 18 months of new recruitment, highlighting which proved most effective and cost-efficient in order to inform future research.

**Objective:**

This study evaluated recruitment strategies using four main outcomes: (1) absolute recruitment numbers, (2) sociodemographic representativeness, (3) biosample return rate, and (4) cost per participant.

**Methods:**

Between May 2022 and December 2023, recruitment was undertaken via snowball recruitment (through friends and family of existing volunteers), invitations to those who participated in a previous survey (CovidLife: the GS COVID-19 impact survey), and Scotland-wide recruitment through social media (including sponsored Meta-advertisements), news media, and TV advertisement. The method of recruitment was self-reported in the baseline questionnaire. We present absolute recruitment numbers and sociodemographic characteristics by recruitment method and evaluate the saliva sample return rate by recruitment strategy using chi-square tests. The overall cost and cost per participant were calculated for each method.

**Results:**

In total, 7889 new participants joined the cohort over this period. Recruitment sources by contribution were social media (n=2436, 30.9%), survey responder invitations (n=2049, 26.0%), TV advertising (n=367, 17.3%), snowball (n=891, 11.3%), news media (n=747, 9.5%), and other methods or unknown (n=399, 5.0%). More females signed up than males (5570/7889, 70.5% female). To date, 83.5% (6543/7836) of participants returned their postal saliva sample, which also varied by demographic factors (3485/3851, 90.5% older than 60 years vs 471/662, 71.1% aged 16‐34 years). Average cost per participant across all recruitment strategies was £13.52 (US $16.82). Previous survey recontacting was the most cost-effective (£0.37 [US $0.46]), followed by social media (£14.78 [US $18.39]), while TV advertisement recruitment was the most expensive per recruit (£33.67 [US $41.89]).

**Conclusions:**

This study highlights both the challenges and the opportunities in large web-based cohort recruitment. Overall, social media advertising has been the most cost-effective and easily sustained strategy for recruitment over the reported recruitment period. We note that different strategies resulted in successful recruitment over varying timescales (eg, consistent sustained recruitment for social media and large spikes for news media and TV advertising), which may be informative for future studies with different requirements of recruitment periods. Limitations include self-reported methods of recruitment and difficulties in evaluating multilayered recruitment. Overall, these data demonstrate the potential cost requirements and effectiveness of different strategies that could be applied to future research studies.

## Introduction

### Background

Recruitment of participants to population-based health research studies is a major challenge. Web-based recruitment strategies, including social media, are increasingly used for their reported low cost, time effectiveness, and potential wide reach [1-5] while also complementing the increasing prevalence of web-based data collection, such as web-based questionnaires. Social media is reported to be particularly advantageous for targeting specific populations through demographic filters and location settings. Despite these advantages, the evidence on the effectiveness of remote recruitment across sociodemographic groups is mixed. While social media platforms have been reportedly successful in recruiting young people [6,7] and hard-to-reach populations [8,9], other studies report challenges in recruiting men [10,11] and older adults [4,12].

Web-based strategies are often combined with more traditional advertising media such as newspapers, radio, TV, and email [13-18], offering a multimodal approach to recruitment that aims to optimize reach and demographic coverage. Several studies have reported that a combined approach can lead to more effective campaigns by enhancing coverage, improving trust, and increasing diversity [15,19]. It has also been reported that traditional methods observe higher participation completion rates [20,21], an important consideration for studies requiring sustained participant engagement, such as longitudinal data collections or biosample returns. There is also growing recognition of the privacy, confidentiality, and informed consent concerns surrounding social media recruitment [22,23]. Participants may perceive social media as a less trustworthy recruitment source than more established offline channels.

Few population-based studies have published detailed comparisons of different recruitment methods, in terms of recruitment yields, cost-effectiveness, and sociodemographic reach by these different methods. In the context of a longitudinal population-based cohort, Generation Scotland (GS), we therefore describe different strategies and compare in terms of recruitment effectiveness parameters. Most existing studies focus on specific subpopulations or disease groups, limiting the ability to generalize findings to broader cohorts. GS provides an opportunity to examine recruitment in the context of a broad (anyone in Scotland aged 12+ years) and large (20,000 participants) recruitment target, which allows comparison of recruitment effectiveness across demographic factors. We also report on a unique recruitment method, as to our knowledge, no other health study has used paid television advertising to recruit participants. In addition, we examine the success of remote biosampling return rates by methods of recruitment, which is relatively underexplored in the literature. Another key gap in the literature is the limited evidence on how recruitment effectiveness varies over time. We explore relative comparison of effectiveness over an extended recruitment time period (20 months) and discuss the synergistic impact of combined methods in a multilayered strategy.

### GS Cohort Background

GS is a large Scottish family health cohort, established as a resource to support research on the genetic and environmental determinants of mental and physical health and well-being. The original cohort was established between 2006 and 2011 and was made up of around 24,000 individuals across Scotland, aged 18+ years [24]. The mean age at recruitment was 47 (SD 15) years, and the cohort comprises 59% females. Participants in the original cohort were recruited using a general practice–based recruitment strategy by postal and telephone contact, with in-person clinic attendance (89%) or postal sample and remote questionnaire collection (11%) at considerable amount of staff time and postal charges [24].

In 2019, funding was obtained to expand the cohort and recruit an additional 20,000 individuals [25]. In the new phase of recruitment, all data, questionnaires, and biosamples (saliva) are collected remotely. This format allows individuals to sign up at a convenient time and from any location without having to travel to a clinic, minimizing the cost and effort required to participate. As a population-based study across Scotland, we considered it important to remove barriers of participation for underrepresented groups, for example, individuals from rural communities and those who find travel difficult. We also lowered the age of eligibility to join to 12+ years in order to support health research in the younger generation of individuals living in Scotland. Note that this paper focuses on recruitment of individuals aged 16+ years, with 12‐ to 15-year-old recruitment being the subject of another paper. Teen-specific recruitment strategies were applied to this age group, with these strategies still being assessed. Postal saliva collection was selected as the biosampling measure as it performs well in DNA extraction and genetic analysis [26] and was considered appropriate for remote sampling and more acceptable than alternatives such as blood sampling, which was required in the original cohort. Recruitment for the expansion of the cohort began in May 2022 and has since used a range of approaches, traditional and web-based, within a multilayered strategy. Here, we report strategies used in the recruitment of new participants to the GS cohort up to the end of December 2023.

### Objective

The primary aim of this paper is to evaluate a range of strategies used to recruit new participants to the GS cohort using four main outcomes: (1) absolute recruitment numbers, (2) sociodemographic representativeness, (3) biosample return rate, and (4) cost per participant. Reports of demographic and geographic reach can be used to inform recruitment strategies, timelines, and costs required for future studies.

## Methods

### Study Design

The GS study aims to expand the original cohort (N=24,000) by recruiting an additional 20,000 new participants across Scotland. Although to date, recruitment is ongoing, this report focuses on a comparison of recruitment methods used during the initial main phase of population recruitment, conducted from May 2022 to the end of December 2023.

Participants interested in the study sign up online on the GS web portal [27]. This online platform takes volunteers through participant information and cohort consents and provides access to baseline and follow-on questionnaires, as well as prompting a request for an address to post the saliva testing kit. Since we have lowered our recruitment eligibility age to anyone living in Scotland aged 12 years and older, we have implemented additional levels of confirmation of their capacity for consent for those aged 12‐15 years by their parent or guardian. This report, however, focuses on participants aged 16 years or older since different recruitment methods are being used for participants younger than 16 years and will be reported separately.

To assess recruitment routes, this study used responses to the baseline survey question: “How did you hear about GS?” Participants were asked to select 1 option from a predefined list itemized in Textbox 1. If a participant chose “Other,” they could provide additional details via a free text response. These responses were the primary method for assigning recruitment routes. Sign-up dates were also taken into account, for example, TV was assigned only as a route if TV was selected after the June 5, 2023, when the TV advertisement began. Email records were additionally used to assign the CovidLife invitation route if the questionnaire response was not completed but individuals were known to have received a CovidLife email invitation. Full definitions for assigning recruitment method are detailed in Table S1 in Multimedia Appendix 1.

In general, we consider that a person is formally a full member of the GS cohort if they have completed the study consent, baseline survey, and returned their saliva sample for genetic analysis. However, in order to be able to report on the saliva sample return rate, a successful recruitment, and therefore the inclusion criteria for this study, was defined as an individual who had completed the study consent and baseline survey only. In total, 840 individuals were excluded for not completing the baseline survey.

Textbox 1. **Baseline questionnaire response options for the question “How did you hear about GS?” (only 1 option allowed; GS: Generation Scotland).**

How did you hear about Generation Scotland?

1. Through a family member
2. Through a friend
3. Employer or organization
4. Social media
5. Radio
6. TV
7. Newspaper or online news source
8. Other organization, for example, charity or other study
9. Clinician or general practitioner
10. Contacted by Generation Scotland after taking part in a previous study, for example, CovidLife
11. Generation Scotland website
12. Web search (option removed in December 2023)
13. The Scottish Health Research Register (SHARE) [28] Newsletter (option added in December 2023)
14. Public engagement event (option added in December 2023)
15. College or university (option added in December 2023)
16. School (option added in December 2023)
17. Poster/billboard/flyer (option added in December 2023)
18. Other (add free text)

### Recruitment Methods

**Figure 1.**
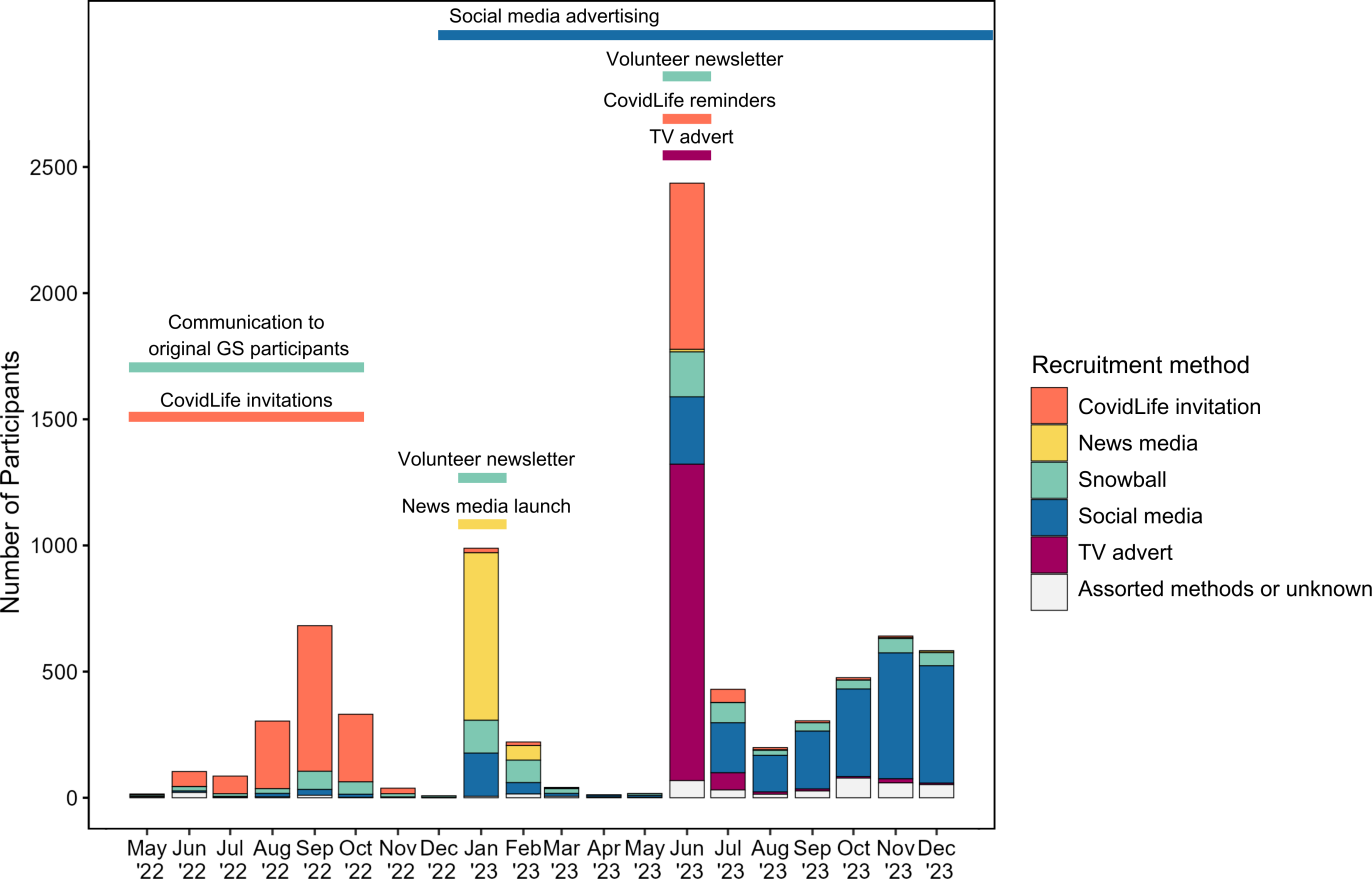
Timeline of new participant recruitment to the Generation Scotland cohort from May 2022 to December 2023, including the periods of coverage for each recruitment method activity. GS: Generation Scotland.

Recruitment began in May 2022, and this study reports activities to the end of December 2023. A “soft launch” of the cohort was conducted between May and December 2022 to trial the performance of the GS portal with limited numbers and to use our existing network of volunteers to recruit new participants. From January 2023, we pursued additional, Scotland-wide recruitment methods, including a news media launch, social media advertising, and a TV advertisement. This took inspiration from the multilayered approach of the Genetic Links to Anxiety and Depression study [13]. Figure 1 shows a timeline of recruitment activities and corresponding sign-up numbers.

#### Snowball Recruitment (May-October 2022 and January and June 2023)

Snowball recruitment, where research volunteers were asked to recommend joining GS to relatives, friends, and other contacts, was the first strategy adopted. From May to October of 2022, members of the original GS cohort with an available email address were invited to join the online portal themselves and at the same time encouraged to invite additional friends and family members to join (n=9531).

In late January 2023, a newsletter was sent to all GS participants providing a further update on the expansion of the cohort and again encouraging existing participants to invite friends and family members to join. The newsletter was sent to 7916 individuals by email, including original GS participants and new sign-ups. Paper newsletters were sent to original GS cohort participants with a postal address but no email information available (n=11,642). Another email newsletter was sent in June 2023 to 6056 participants who had so far joined the online portal.

#### Invitation to Previous Survey Responders: CovidLife (May-December 2022 and June 2023)* *

CovidLife was an observational study run by GS to investigate the impact of the COVID-19 pandemic on mental health and well-being of people living in the United Kingdom [29]. Approximately 18,000 members of the UK population took part (92.2% resided in Scotland). Recruitment to the CovidLife study took place from March 2020 and was mostly achieved through emails to participants of the National Health Service (NHS) Scottish Health Research Register (SHARE) [28]. Individuals living in Scotland who took part in CovidLife and consented to being recontacted were invited to join GS by email from May to December 2022 (n=13,262). In June 2023, a further 11,355 reminder emails were sent to individuals who had not responded to the first invitation.

#### News Media (January 25, 2023)

On January 25, 2023, following the success of the soft launch, wider recruitment was launched with a media campaign composed of traditional media (TV news, radio, and newspaper) and social media (Twitter, Facebook, and Instagram) strategies. A variety of media coverage, including online news articles [30-33] and TV and radio news slots, was arranged for the launch date. This included a front-page BBC (British Broadcasting Corporation) Scotland web article and live question and answer interview on BBC Scotland. There were also news features on The University of Edinburgh and University of Aberdeen websites.

#### Social Media (January 2023 to December 31, 2023)

Social media adverts were run on Meta (Facebook and Instagram) by the GS team from January 2023. Spirit Media [34], a media marketing and advertising agency spun out from The University of Edinburgh, has assisted with developing a social media strategy using sophisticated audience targeting since June 2023. Approximately 4,840,503 impressions (number of views) have been received for Meta from June to the end of December 2023. In addition to advertising, the GS Twitter, Facebook, LinkedIn, and Instagram accounts have been actively posting information about GS and lay summaries to highlight research facilitated by the GS cohort. Approximately 3 posts per week have been produced since May 2023.

#### TV Advertising (June 5-30, 2023)

A TV advertisement was run in June 2023 on Scottish Television commercial channel [35]. The 30-second advertisement featured 3 current GS volunteers sharing their experiences of taking part in GS. The advert was designed to show the ease and potential impact of joining the cohort. It ran for 25 days (June 5-30), for an average of 6 slots per day, at 70% daytime and 30% peak times with an estimated 11,866,880 impressions.

#### Assorted Methods

Emails and letters were sent to members of other research studies including parents and children of the Growing Up in Scotland study [36], and GS was featured in various newsletters, including the SHARE [28], Aberdeen Children of the 1950s [37], and Dyslexia Scotland [38]. Members of the GS team have attended events, including the Edinburgh Science Festival, Glasgow Science Festival, and the Royal Highland Show. Flyers were distributed at these events with QR codes directing individuals to the GS portal. From October 2023, general practices that were part of the Primary Care Research Network began contacting eligible registered individuals by text, inviting them to join GS. These recruitment methods are not included in detail in this study as the overall recruitment numbers were relatively low (total assorted methods, n=391), were not active during the full study period for this analysis, and relevant options for some of these assorted methods were added only to the baseline survey question “How did you hear about GS?” in December 2023. However, we consider these activities important for awareness raising and communicating the importance of health research to the public.

### Recruitment Effectiveness Metrics

Sociodemographic characteristics of recruited participants are presented for each recruitment method compared with the general Scottish population to assess representativeness of recruits from each strategy. This included comparisons of age, sex, ethnicity, local authority regions, Scottish Index of Multiple Deprivation (SIMD) quintiles, and urban-rural classifications. The response rate is presented for the CovidLife survey responders, TV, and social media methods. This was calculated for each method by dividing the total sign-ups per method by the estimated number of impressions (TV and social media) or the total number of emails sent (CovidLife responder invitations). For snowball recruitment and news media, it was not possible to determine a denominator.

We also evaluated the saliva sample return rate using chi-square tests to test the association between recruitment method and demographic factors [39]. Post hoc pairwise tests using Bonferroni adjustment were applied for multiple comparisons [40]. The overall cost and cost per participant of each method are presented. Supplies and service costs and human resource costs were estimated to determine the time required to carry out and sustain each strategy. Time effectiveness of recruitment methods and the overall impact of implementing a multilayered recruitment strategy were not formally measured in this study; however, key observations and practical insights related to these aspects are discussed.

### Ethical Considerations

This study involves human participants. All components of GS received ethical approval from the NHS Tayside Committee on Medical Research Ethics (REC reference number 05/S1401/89). GS has also been granted Research Tissue Bank status by the East of Scotland Research Ethics Service (REC reference number 20-ES-0021), providing ethical approval for a wide range of uses within medical research. Written informed consent was obtained from all participants recruited to the original Generation Scotland: Scottish Family Health Study. Next Generation Scotland–recruited participants gave consent on the web. All participants aged 16 years or older provide their own informed consent, while those aged 12‐15 years require parental confirmation of their capacity to consent. Participants gave informed consent to participate in the study before taking part. Participants included in this study did not receive compensation. All GS study data are deidentified, assigned a unique study ID, and stored separately from any personally identifiable information. Only approved research team members have access to identifiable information.

## Results

### Recruitment Method Effectiveness

In total, 7889 new participants who had completed study consents and the baseline questionnaire were recruited to GS in the study period. Figure 1 shows the timeline of recruitment activities and corresponding sign-up numbers. For 399 (399/7889, 5.0%) individuals, the recruitment method was assigned as either unknown or one of the described assorted recruitment methods.

#### Snowball Recruitment

From May to December 2022, emails were sent to original GS participants encouraging snowball recruitment. Overall, 891 individuals were recruited through invitation from a friend or a family member with upticks coinciding with the January and June 2023 newsletters (Figure 1).

#### Invitation to Previous Survey Responders: CovidLife

Email invitations to 13,262 CovidLife participants, sent from May to December 2022, resulted in recruitment of 1306 individuals (9.8% response rate and 16.6% of total recruits). Reminder emails were sent in June to 11,355 individuals, resulting in a further 743 sign-ups (6.5% response rate to reminders and 9.4% of total recruits).

#### News Media

Recruitment across Scotland via a variety of media coverage resulted in 526 sign-ups (526/7889, 6.7% of total recruits) on launch day (January 25, 2023) and 747 (747/7889, 9.5%) sign-ups from news media in total.

#### Social Media

Social media adverts generated the most sign-ups, 2436 individuals (2436/7889, 30.9%) to the end of December 2023. This equates to an average of 312.5 sign-ups per month since Spirit Media started coordinating social media recruitment in June 2023 to the end of December 2023. Given a total impressions of 4,840,503 across Meta, response rate to social media advertising is an estimated 0.05% (2436/4,840,503).

#### TV Advertising

The June TV advert led to the recruitment of 1367 participants, 17.3% (1367/7889) of the recruitment total. This was achieved within a single month of the advert running. The TV advert received an estimated 11,866,880 impressions resulting in a response rate of 0.012% (1367/11,866,880).

### Sociodemographic Characteristics

Sociodemographic characteristics of the 7889 new participants are presented by recruitment strategy in Table 1. Overall, there was a high sex imbalance with more females recruited to the cohort (5570/7889, 70.6% female). Study participants were older, with 49.1% (3874/7889) of the participants older than 60 years compared with 30.8% of the Scottish population. Among newly recruited participants, 1.2% (92/7889) were of non-White ethnicity compared with 7.1% of the Scottish population. Generally, new sign-ups were living in less deprived regions compared with the general population; however, new recruits were relatively well matched for regional representation (defined according to local authority regions) and for urban-rural classifications.

Comparing recruitment methods, social media had the greatest sex imbalance with 80.0% (1950/2436) of recruits identifying as female. Snowball recruitment and news media methods were the most successful at recruiting younger adults with 21.2% (189/891) and 13.3% (99/747) of recruits aged 16‐34 years, respectively. Previous survey respondents (CovidLife) had the highest proportion of older recruits (1269/2049, 61.9% aged older than 60 years), followed by social media (1207/2436, 49.5% aged older than 60 years). A TV advertisement demonstrated the best match of Scottish population distribution across SIMD quintiles, with 16.5% (226/1367) of TV recruits in the most deprived SIMD quintile. Those recruited via social media also had a relatively even population spread within SIMD quintiles (598/2436, 24.7% to 291/2436, 11.9%).

**Table 1.** Summary of demographic characteristics by recruitment method with comparison to the Scottish population.

	TV	News media	Social media	CovidLife invitation	Snowball recruitment	Total	Scotland population (%)
Total participants, n (%)	1367 (17.3%)	747 (9.5%)	2436 (30.9%)	2049 (26.0%)	891 (11.3%)	7889^a^	N/A^b^
Sex							
Female	937 (68.5%)	453 (60.6%)	1950 (80.0%)	1386 (67.6%)	555 (62.3%)	5570 (70.6%)	51.9^c^
Male	428 (31.3%)	293 (39.2%)	483 (19.8%)	662 (32.3%)	334 (37.5%)	2309 (29.3%)	48.1^c^
Missing/prefer not to say	<10	<10	<10	<10	<10	10 (0.1%)	N/A
Age (years)							
16‐34	86 (6.3%)	99 (13.3%)	174 (7.1%)	64 (3.1%)	189 (21.2%)	669 (8.5%)	28.1^c^
35‐60	652 (47.7%)	363 (48.6%)	1055 (43.3%)	716 (34.9%)	358 (40.2%)	3346 (42.4%)	41.1^c^
>60	629 (46.0%)	285 (38.2%)	1207 (49.5%)	1,269 (61.9%)	344 (38.6%)	3874 (49.1%)	30.8^c^
Ethnicity							
White	1358 (99.3%)	731 (97.9%)	2,412 (99.0%)	2016 (98.4%)	881 (98.9%)	7783 (98.7%)	92.9 ^d^
Other	<10	16 (2.1%)	21 (0.9%)	27 (1.3%)	<15	92 (1.2%)	7.1^d^
Missing	<10	<10	<10	<10	<10	14 (0.2%)	N/A
Scotland region							
Aberdeen and North East	177 (12.9%)	81 (10.8%)	299 (12.3%)	415 (20.3%)	110 (12.3%)	1122 (14.2%)	10.7^c^
Edinburgh and Lothians	212 (15.5%)	202 (27.0%)	420 (17.2%)	693 (33.8%)	233 (26.2%)	1886 (23.9%)	16.7^c^
Glasgow and Strathclyde	527 (38.6%)	211 (28.2%)	694 (28.5%)	382 (18.6%)	208 (23.3%)	2124 (26.9%)	40.7^c^
Highland and Islands	127 (9.3%)	71 (9.5%)	280 (11.5%)	144 (7.0%)	79 (8.9%)	731 (9.3%)	7.2^c^
Scotland South	<10	<30	165 (6.8%)	99 (4.8%)	40 (4.5%)	355 (4.5%)	4.8^c^
Tayside, Central and Fife	310 (22.6%)	155 (20.7%)	557 (22.9%)	304 (14.8%)	191 (21.4%)	1594 (20.2%)	19.9^c^
Missing	<15	<10	18 (0.7%)	12 (0.6%)	31 (3.5%)	79 (1.0%)	N/A
SIMD^e^							
1: Most deprived	226 (16.5%)	58 (7.8%)	291 (11.9%)	83 (4.1%)	57 (6.4%)	757 (9.6%)	20.6 ^f^
2	256 (18.7%)	104 (13.9%)	393 (16.1%)	237 (11.6%)	113 (12.7%)	1161 (14.7%)	21.1^f^
3	291 (21.3%)	143 (19.1%)	528 (21.7%)	347 (16.9%)	167 (18.7%)	1550 (19.6%)	19.8^f^
4	306 (22.4%)	184 (24.6%)	608 (25.0%)	584 (28.5%)	219 (24.6%)	2004 (25.4%)	19.3^f^
5: Least deprived	278 (20.3%)	257 (34.4%)	598 (24.5%)	786 (38.4%)	304 (34.1%)	2338 (29.6%)	19.2^f^
Missing	<15	<10	18 (0.7%)	12 (0.6%)	31 (3.5%)	79 (1.0%)	N/A
Urban-rural							
1: Large urban areas	420 (30.7%)	287 (38.4%)	651 (26.7%)	786 (38.4%)	336 (37.7%)	2644 (33.5%)	37.8^g^
2: Other urban areas	541 (39.6%)	189 (25.3%)	788 (32.3%)	412 (20.1%)	225 (25.3%)	2253 (28.6%)	33.9^g^
3: Accessible small towns	105 (7.7%)	60 (8.0%)	236 (9.7%)	179 (8.7%)	63 (7.1%)	675 (8.6%)	8.6^g^
4: Remote small towns	35 (2.6%)	18 (2.4%)	116 (4.8%)	63 (3.1%)	15 (1.7%)	259 (3.3%)	2.6^g^
5: Accessible rural areas	182 (13.3%)	115 (15.4%)	392 (16.1%)	438 (21.4%)	131 (14.7%)	1319 (16.7%)	11.6^g^
6: Remote rural areas	74 (5.4%)	77 (10.3%)	235 (9.6%)	159 (7.8%)	90 (10.1%)	660 (8.4%)	5.5^g^
Missing	<15	<10	18 (0.7%)	12 (0.6%)	31 (3.5%)	79 (1.0%)	N/A

a Total includes 399 individuals where recruitment method was assigned as an “assorted recruitment method/unknown.”

b N/A: not applicable.

c Based on National Records of Scotland (NRS) Mid-2023 Population Estimates [41]. Restricted to those aged 16+ years.

d Based on NRS 2022 Scottish Census [42].

e SIMD: Scottish Index of Multiple Deprivation.

f Based on 2021 NRS Population Estimates by Scottish Index of Multiple Deprivation (SIMD) [43]. Restricted to those aged 16+ years and estimated from SIMD deciles.

g Based on the 2021 NRS Scottish Government Urban Rural Classification [44]. Restricted to those aged 16+ years.

### Saliva Sample Return Rate

After completing the study consents and baseline questionnaire, participants were sent a postal saliva sample kit. Reminders were sent to individuals to return their saliva sample kit at 3, 6, and 10 weeks. Median time to return a saliva sample was 13 (IQR 8‐21) days. Overall, 83.5% (6543/7836) of participants returned their postal saliva sample (Table 2). The sample return rate was compared across recruitment methods and demographic factors, full chi-square results, and pairwise comparisons are provided in Multimedia Appendix 2.

A chi-square test was performed to examine the association between recruitment method and saliva sample return rate and indicated a statistically significant difference across recruitment methods (*χ*²_5_ [n=7836]=148.83; *P*<.001). Post hoc pairwise comparisons with Bonferroni correction identified that participants recruited via the CovidLife survey had the highest return rate (1840/2036, 90.4%), which was statistically significantly higher (*P*<.001) than the return rates for participants recruited via snowball recruitment (738/889, 83.0%), social media (1923/2416, 79.6%), and TV advertising (1068/1360, 78.5%).

Males had a significantly higher saliva sample return rate (2040/2298, 88.8%) than females (4495/5528, 81.3%), as indicated by a chi-square test (*χ*²_1_ [n=7826]=65.03; *P*<.001). Comparing the return rate among age groups, a chi-square test showed a statistically significant difference (*χ*²_2_ [n=7836]=287.06; *P*<.001). Post hoc pairwise comparisons with Bonferroni correction revealed that individuals older than 60 years had a significantly higher saliva sample return rate (3485/3851, 90.5%) than those aged 16‐34 years (471/662, 71.1%; *P*<.001). In addition, saliva sample return rates differed significantly across deprivation quintiles (*χ*²_4_ [n=7798]=151.96; *P*<.001). Post hoc pairwise comparisons with Bonferroni correction indicated that participants from the least deprived areas (2071/2336, 88.7%) had significantly higher return rates than those from the most deprived areas (540/755, 71.5%; *P*<.001).

**Table 2.** Summary of saliva sample return rate following sign-up, by recruitment method and demographic factors^a^.

	Saliva sample return (n=7836), n (%)
Total	6543 (83.5)
Recruitment method	
CovidLife invitation	1840 (90.4)
News media	668 (89.7)
Snowball recruitment	738 (83.0)
Social media	1923 (79.6)
TV	1068 (78.5)
Other	306 (78.5)
Sex	
Female	4495 (81.3)
Male	2040 (88.8)
Age group (years)	
16‐34	471 (71.1)
35‐60	2587 (77.9)
>60	3485 (90.5)
Ethnicity	
White	6460 (83.6)
Other	73 (79.3)
SIMD^b^	
1: Most deprived	540 (71.5)
2	909 (78.4)
3	1279 (82.8)
4	1711 (85.5)
5: Least deprived	2071 (88.7)

a Only those individuals who were sent a specimen kit (excludes those with no address information, n=53) were included i then denominator (N=7836).

b SIMD: Scottish Index of Multiple Deprivation.

### Cost-Effectiveness of Recruitment Strategies

Excluding individuals recruited from assorted methods or unknown (n=7490), the overall cost per participant recruited was £13.52 (US $16.82) across the 5 highlighted methods, with a total spend of £101,261 (US $125,992.37; Table 3). Detailed cost breakdowns are shown in Table S2 in Multimedia Appendix 1. TV was the most expensive (albeit rapid) strategy in both absolute terms and by cost per participant (£33.67 [US $41.89]). The production cost of the high-quality TV-ready advert was £6316 (US $7857.08) while the Scottish Television commercial channel advertising campaign cost £34,650 (US $43,112.60). We note that the advert has been reused repeatedly on social media platforms representing value for money on the production of the initial advert. Snowball recruitment had the second highest cost per recruit at £17.89 (US $22.26). The bulk of the expenditure for this was attributed to posting paper newsletters to original GS volunteers in January 2023 where an email address was not available (£13,794 [US $17,161.61]). The total spend on social media recruitment was £36,003 (US $44,792.97), which equated to £14.78 (US $18.39) per participant recruited. Social media costs included advertisement costs, Spirit Media marketing services, and staff time to create regular social media posts and respond to comments and messages.

The least expensive method was invitations to CovidLife survey responders (£0.37 [US $0.46] per recruitment), which required minimal staff hours to prepare communications and email CovidLife participants. The news media strategy was also low-cost (£3.39 [US $4.22] per recruitment), which took advantage of The University of Edinburgh’s central media and press office services but otherwise using unpaid media sources.

**Table 3.** Cost-effectiveness of recruitment methods^a^.

Recruitment method	N	Cost (US $)	Cost per recruitment (US $)
TV	1367	£46,025.20 ($57,271.35)	£33.67 ($41.89)
Social media	2436	£36,003.35 ($44,792.97)	£14.78 ($18.39)
Snowball recruitment	891	£15,944.33 ($19,838.54)	£17.89 ($22.26)
News media	747	£2529.80 ($3147.58)	£3.39 ($4.22)
CovidLife invitation	2049	£758.94 ($944.30)	£0.37 ($0.46)
Total	7490^b^	£101,261.62 ($125,992.37)	£13.52 ($16.82)

a Detailed cost breakdowns are shown in Table S2 in Multimedia Appendix 1.

b Total excludes the 399 individuals where recruitment method was from an “assorted recruitment method or unknown.”

## Discussion

### Recruitment Effectiveness

This study reports the effectiveness of a variety of strategies used in the recruitment of individuals to the GS longitudinal cohort. A multilayered approach was taken, using a combination of recruitment methods, leading to the recruitment of 7889 new participants. We report data to the end of December 2023; however, recruitment is currently ongoing, with the aim to conclude in June 2025.

Table 4 provides an overall assessment of the recruitment strategies used. The initial recruitment plan was to exclusively use existing GS volunteers to drive snowball recruitment. This was intended to build upon the existing family aspect of the cohort. Overall, snowball recruitment cost £17.89 (US $22.26) per new volunteer, driven by postage expenses for the annual newsletter. Future studies should be mindful of reliance on existing networks of volunteers to drive recruitment, especially when initial recruitment occurred a substantial time ago (original GS recruitment was 2006), and it becomes more challenging to maintain general engagement and up-to-date contact information for participants.

In contrast, invitation to previous survey responders (CovidLife) by email was a highly successful strategy for overall recruitment numbers, relatively inexpensive, and required minimal staff time. In contrast to the snowball recruitment, this method yielded a high response rate (1306/13262, 9.8%), likely because it targeted a population more recently engaged in health research and for whom current email contact information was available. Although this was an effective strategy, it was reliant on an existing pool of volunteers and therefore did not reach a new audience and could target only a finite number of individuals. There were also concerns that repeated messaging to potential volunteers might have limited repeated returns or even incurred a net negative impact limiting the number of times this strategy could be used.

Because of its nationwide reach, the media launch was successful in gaining a substantial number of recruits within a few days, but sign-ups dropped sharply thereafter. This is likely due to the short-lived nature of media coverage. This strategy reached a wide audience and used potentially trustworthy news sources. Similarly, the TV advert received more than 11 million impressions and reached a wide audience across Scotland. These strategies demonstrated the value of a multilayered approach as social media–derived recruitment was also increased in January (concurrent with the news coverage), compared with February and March, and during the June TV advert campaign compared with July and August. The TV advert was highly successful in driving many recruits in a short time period; however, this strategy was expensive and not affordable over a longer period (Table 4). The TV advert was also repurposed for use as a social media advert beyond the TV advertising and therefore fed into other methods of recruitment. Both the media launch and the TV advert were good strategies for rapid recruitment, and the use of these traditional media is thought to have contributed to an overall increased public awareness and legitimacy with an amplifying impact on the multilayered strategy.

**Table 4.** Summary of strengths and weaknesses of each recruitment method used in the recruitment of participants to the Generation Scotland cohort.

Recruitment method	Strengths	Weaknesses
Snowball recruitment	Cost-effective to use existing network of volunteers We already communicate with volunteers regularly about cohort updates Recruitment within families enhances genetic power of the cohort	Do not want to overtask current volunteers Sending paper letters is expensive compared with emailing volunteers
CovidLife invitation	High response rate Reaches audience already interested in taking part in research Inexpensive to email individuals	Recruiting from participants already taking part in research may reinforce current sociodemographic trends Finite number of individuals to contact
Social media	Cost-effective to run for a long period of time Takes individuals to the sign-up page in one click Reaches wide audience Potential to reach younger individuals	Demographic selection bias toward women (algorithm feedback) Requires considerable time investment to maintain online presence Lack of control over platform algorithms
News media	Reaches wide audience Uses potentially trustworthy news sources	Very short term (eg, a news article will be front page only for 1 day) Requires contacts to reach news outlets Limited control over materials produced
TV advertising	Reaches wide audience Rapid recruitment Broad SIMD^a^ recruitment Reusability of TV advert for social media	Expensive to produce TV-ready advert Expensive to run an advert campaign

a SIMD: Scottish Index of Multiple Deprivation.

Social media engagement and advertising have been active since February 2023 and have generated steady sign-ups overall, bringing in the highest number of recruitments. Our observations are consistent with other studies which have demonstrated the benefits of social media recruitment [12,45-47]. Social media has the advantage of immediacy in directing individuals to sign up with a single click while already using an online device. Algorithm-driven advertising directs the adverts to those most likely to sign up and has the ability to target a desired audience, including age range and location. This method also offers greater control of advertising expenditure than other recruitment routes and has been effective as a longer-term sustainable strategy.

### Sociodemographic Characteristics

Overall, we found a gender imbalance across all recruitment methods with a higher proportion of female recruits (5570/7889, 70.6% females) and also a higher proportion of older individuals (3874/7889, overall 49.1% aged older than 60 years compared with 30.8% in the Scottish population). We report social media to be the most female-biased method and have so far recruited only small numbers of younger participants (aged 16‐34 years) through this method. This finding contrasts with other studies that have reported social media as being effective at reaching younger populations [12,48,49]. This may be due to advertising materials (ie, the more traditional TV advert) appealing more to an older demographic. Demographic patterns may also be reinforced by the Meta algorithm pushing adverts to those with similar characteristics to those who have previously clicked on an advert [50].

Low ethnic diversity (92/7889, 1.2% non-White vs 7.1% in the Scottish population) was reported across all strategies, consistent with existing trends in research participation [51-55], and other similar cohorts such as the Avon Longitudinal Study of Children and Parents (2.2% non-White mothers) [56] and UK Biobank (5.4% non-White participants) [57]. The lack of differential success across strategies suggests that the methods and materials applied may not adequately reach or resonate with ethnic minority populations. This highlights the need for further work to address barriers to participation [58]. Although we adopted inclusive practices recommended in recent literature, such as featuring diverse representatives in our recruitment materials [59], evidence shows that meaningful increases in participation often require resource-intensive, community outreach approaches. Indeed, had we relied more heavily on snowball recruitment, as initially planned, diversity may have been even lower. More outreach-based approaches, such as events and school engagement, were undertaken to recruit 12‐ to 15-year-olds to the GS study (to be reported separately), which achieved greater diversity (11.5% non-White participants) and may help inform how future studies continue to address this issue.

Although overall participants recruited to the cohort were skewed toward individuals of relatively high socioeconomic status, the TV advert notably recruited individuals from a broader sociodemographic range than is often achieved in research studies. This was also observed by the NutriNet-Santé study (in France), which also used TV advertising as a recruitment strategy [60]. This highlights the potential value of incorporating mass media approaches to reduce sociodemographic biases and improve representativeness.

Participants recruited to the cohort were well aligned with the Scottish population in terms of rural representation. This likely reflects the use of remote, geographically unrestricted, and broadly targeted recruitment methods, along with a web-based sign-up process. Despite challenges in achieving proportional representation, a common concern among similar studies, we have achieved good geographic reach. The expansion of the GS cohort to more than 40,000 individuals has increased overall diversity and introduced a broader range of exposures and confounders, thereby enhancing the cohort’s value for investigating associations between exposures and health outcomes.

### Saliva Sample Return Rate

Although overall postal saliva sample return is good, at more than 83% (6543/7836), our study highlights the requirement to oversample by 20% to obtain the desired biosample target. Those recruited via TV advertising (1068/1360, 78.5%) and social media (1923/2416, 79.6%) had the lowest saliva sample return rates, although these rates remained relatively high overall. A chi-square test indicated a statistically significant difference in sample return rates across recruitment methods (*P*<.001), and post hoc pairwise comparisons confirmed that return rates for both TV and social media were significantly lower than those for CovidLife responders (*P*<.001), the method with the highest saliva sample return rate (1840/2036, 90.4%). This was expected given TV and social media strategies had the broadest recruitment target, reaching harder-to-recruit demographics and individuals less connected to the project than CovidLife previous survey responders. Future studies may consider the potential lower returns of mass reach recruitment strategies and build in oversampling or additional follow-up mechanisms.

Sample return was also lower in some demographic groups, including younger people (471/662, 71.1% aged 16‐34 years), although still overall at relatively high levels. This highlights the need for additional recruitment efforts in these groups, with specific recruitment strategies, to reach sample target numbers across a diverse demographic.

### Ongoing Development

We aimed to address barriers to recruitment with ongoing development of the sign-up process. We provided email and phone assistance to all participants to answer any queries about the cohort or assist in the sign-up process. Adjustments have been made through the recruitment period based on volunteer feedback to improve our recruitment process. This included the creation of a landing page (the web page an individual “lands” on after following a link) [27] to improve user experience. The option to sign up using a phone number and receive updates via SMS text messaging instead of an email address was also added with the aim of improving accessibility, particularly for young people, based on feedback from our Young Persons Advisory Group and parents. Since its introduction in June 2023, 15% (769/5084) of volunteers included in this study have signed up primarily using a mobile number.

### Withdrawal From GS

Participants are able to withdraw from the study at any time to remove their data and have their sample destroyed. The average withdrawal rate for the cohort during the recruitment period was 0.44% (38/8631) in line with other similar studies [17,28].

### Study Strengths and Limitations

The strengths of the study are the reporting of novel approaches used in the remote recruitment of participants to a national cohort (Table 4). While other studies such as the NutriNet-Santé study [60] and Genetic Links to Anxiety and Depression study [13] have reported use of TV recruitment through news reporting and media coverage, to our knowledge, no other health study has used a paid TV advertisement to recruit participants. In addition, we report important evidence on the effectiveness of social media. In the literature, social media is often reported to be most effective when targeting specific hard-to-reach populations [49,61-63] or younger individuals [64,65]. Here, we report recruitment with broad inclusion criteria and a large recruitment target. Furthermore, other studies have reported the impact of demographic factors on remote saliva sample return [66,67] but to our knowledge, no other studies have reported how web-based and traditional recruitment methods impact sample return rates.

In this study, we successfully assigned 1 of 5 key recruitment routes to 95% (7490/7889) of new participants using self-reported questionnaire responses. However, a limitation of this study was that self-reported responses may not always be accurate. Participants were able to select only 1 option for “how they heard about GS” when they may have heard about GS from several sources. We do not know whether individuals selected the first source they encountered or the one that motivated the sign-up. Uniquely, for COVIDLife responders, we knew who had been sent a COVIDLife email invitation, providing a second source to determine how they heard about GS. This allowed us to compare questionnaire responses with invitation records. Among previous survey responders known to have received a CovidLife email invite from us, 86.2% (1819/2110) correspondingly selected “COVIDLife invite” while 7.3% (153/2110) reported hearing about GS via social media and 4.1% (87/2110) selected “friend or family.” This indicates that there is overlap between methods and recruitment assignment may be more complex than a single-source question can capture. Future studies should consider allowing participants to self-report multiple sources or rank their influence to improve the accuracy of recruitment pathway analysis. Alternatively, free-text analysis methods could be applied.

Another limitation of the study was the inability to statistically assess the impact of the multilayered recruitment strategy. We can report observational benefits of running multiple recruitment methods at the same time, but it is difficult to disentangle and assess this impact fully.

The cost-effectiveness analysis estimated the hours of staff time required for each recruitment method in order to compare strategies. However, in practice, there have been 15 management and delivery team members with a direct and indirect focus on recruitment activities. Therefore, the true total cost of recruitment would also include salary costs for the team. In addition, historical costs associated with recruitment methods were not taken into account in this study. For example, we report invitations to CovidLife survey responders to be a low-cost strategy. However, we do not take into account the costs associated with recruitment to the CovidLife study in 2020, which required payment to NHS Research Scotland initiative SHARE to email their participants.

### Conclusions

This paper presents some of the challenges and successes of recruitment of individuals to a nationwide cohort. Using a multilayered strategy of recruitment approaches has created a sustained pattern of recruitment, with additional spikes, and has allowed us to reach a broader demographic of participants than with more traditional recruitment methods. In particular, the TV advertising campaign proved highly effective in recruiting a large number of participants in a short amount of time and from a broad sociodemographic background, while social media resulted in the most overall sign-ups over a longer, sustained period. Further efforts are required to improve accessibility with ongoing developments to the sign-up process and appropriate demographic targeting, in particular, to recruit younger individuals. These data demonstrate the potential cost requirements and effectiveness of different strategies that could be considered in the planning of future cohort research studies.

## Supplementary material

10.2196/75116 Multimedia Appendix 1 Full definitions to assign recruitment method to participants, detailed cost breakdowns for recruitment activities, and Generation Scotland management and delivery team summary.

10.2196/75116 Multimedia Appendix 2 Chi-square and pairwise test results evaluating sample return rate between recruitment method and demographic factors.

## Abbreviations

BBC: British Broadcasting Corporation
GS: Generation Scotland
NHS: National Health Service
SHARE: Scottish Health Research Register
SIMD: Scottish Index of Multiple Deprivation
