# Supplementary material for: Effectiveness and Costs of Participant Recruitment Strategies to a Web-Based Population Cohort: Observational Study
Source: J Med Internet Res. 2025 Oct 6;27:e75116. doi: 10.2196/75116 (PMC12500226; doi:10.2196/75116)
Supplement: Multimedia Appendix 1 [file jmir-v27-e75116-s001.docx]

## Multimedia Appendix 1

**Table S1**. Definitions to assign a recruitment method to each new participant using baseline questionnaire responses, free text analysis, sign-up date and records of emails sent to previous survey responders (COVIDLife).

| **Recruitment method** | **Questionnaire Response**  “Where did you hear about GS?” | **Additional Criteria** | |
| --- | --- | --- | --- |
| **Snowball recruitment** | - Through a family member - Through a friend |  | |
| **Social media** | - Social media | **OR** | Free text contains:   - Social media - Facebook - Instagram - Twitter |
| **TV** | - TV | **AND** | Sign-up date ≥05/06/23^c^ |
| **News**  **media** | - Newspaper/online news source - Radio | **OR** | Free text contains:   - News - BBC - **NOT** Newsletter |
|  | - TV - Employer or organisation - Other organisation eg. charity/ other study - Generation Scotland website^a^ - Web search^a^ | **AND** | Sign-up date between  24/01/23-15/02/23^d^ |
| **COVIDLife invite** | - Contacted by Generation Scotland after taking part in a previous study eg. COVIDLife | **AND** | Sign-up date <01/10/23^e^ |
|  |  | **OR** | Received COVIDLife email invite  (If not assigned another method) |
| **Assorted methods** | - Clinician/GP - SHARE Newsletter^b^ - Event^b^ - College/University^b^ - School^b^ - Poster/billboard/flyer^b^ | **OR** | No recruitment method assigned |

^a^ Options removed in December 2023.

^b^ Options added in December 2023.

^c^ Date the TV advert first aired.

^d^ Dates restricted for more general categories that were assumed to be related to the news media launch to three weeks after media launch.

^e^ Date restriction because of contact to individuals through the Growing Up in Scotland (GUS) study and inclusion in the SHARE newsletter from October 2023*.*

**Table S2**. Detailed cost breakdowns for recruitment activities.

| **Recruitment Method** | **Recruitment Activity** | | **Supplies and services cost** | **Staff Hours Description** | **Staff Hours** | **Staff Cost^a^** | **Overall** |  |
| --- | --- | --- | --- | --- | --- | --- | --- | --- |
|  |  |  |  |  |  |  |  |  |
| TV | Advertisement production | | £6,316 | 2 members of staff 1 month | 280 | £5,060 | £5,060 |  |
|  | Advertising with STV | | £34,650 |  |  |  |  |  |
| Social Media | Facebook/ Instagram ads | Newsfeed, story and video ads | £16,000 | 1 member of staff 1 day per month | 49 | £885 | £16,353 |  |
|  |  | Prospecting new audiences, lookalike audience targeting, retargeting content engagers | £1,900 |  |  |  |  |  |
|  | TikTok ads | Newsfeed, story and video ads | £580 |  |  |  |  |  |
|  |  | Prospecting new audiences, lookalike audience targeting, retargeting content engagers | £1,170 |  |  |  |  |  |
|  | Social media posts - average 3x posts per week across Facebook, Instagram, LinkedIn and TikTok. | |  | Jan 23 - Jun 23: 5 hrs/wk Jun 23 - Sep 23: 35 hrs/wk Sep 23 - Dec 23: 7 hrs/wk Dec 23 - Jan 24: 20 hrs/wk | 856 | £15,468 |  |  |
| Snowball | Emails to original GS to invite friends and family to join | |  | 2 members of staff 2 days | 28 | £506 | £2,150 |  |
|  | January 23 newsletters by email | |  | 1 member of staff 2 weeks | 70 | £1,265 |  |  |
|  | January 23 newsletters by post | | £13,794 | 1 member of staff 1 day | 7 | £126 |  |  |
|  | June 23 newsletter by email | |  | 1 member of staff 2 days | 14 | £253 |  |  |
| COVIDLife Invitation | Invitatation emails | |  | 2 members of staff 2 days | 28 | £506 | £759 |  |
|  | Reminder emails | |  | 1 members of staff 2 days | 14 | £253 |  |  |
| News Media | Launch preparation | |  | 1 member of staff 1 month | 140 | £2,530 | £2,530 |  |

^a^ Staff cost calculated using average staff salary at approximately £18.07 per hour (UE06 band 1).

**Table S3**. List of current GS management and delivery team, investigators and members of the Scientific Steering Committee.

| **GS Scientific Steering Committee** |
| --- |
| Professor Dame Anna F Dominiczak (University of Glasgow & Chief Scientist Office, Scotland) – Chair  Professor Cathie Sudlow (University of Edinburgh) – GS Director  Professor Heather Whalley (University of Edinburgh) – GS Chief Scientist  Professor Julie Brittenden (University of Glasgow)  Dr Christian Cole (University of Dundee)  Professor Riccardo Marioni (University of Edinburgh)  Professor Zosia Miedzybrodzka (University of Aberdeen)  Professor Sandosh Padmanabhan (University of Glasgow)  Professor Blair Smith (University of Dundee)  Katie Wilde (University of Aberdeen) |
| **GS Scientific Leadership Team** |
| Professor Cathie Sudlow – GS Director and Principal Investigator (PI)  Professor David Porteous - Co-Investigator (Former Director and PI)  Professor Andrew McIntosh- Co-Investigator  Professor Riccardo Marioni- Co-Investigator  Professor Caroline Hayward- Co-Investigator  Professor Heather Whalley – GS Chief Scientist and Co-Investigator |
| **GS Management and Delivery Team** |
| Professor Cathie Sudlow (University of Edinburgh) – Director  Archie Campbell (University of Edinburgh) - Chief Technology Officer  Robin Flaig (University of Edinburgh) - Chief Operations Officer  Professor Heather Whalley (University of Edinburgh) - Chief Scientist |
| Dr Mark Adams (University of Edinburgh) – Senior Research Fellow  David Buchanan (University of Edinburgh) – Data manager  Fiona Clark (University of Edinburgh) – Partnership Coordinator  Liz Kirby (University of Edinburgh) - Research Support Officer  Hannah Milbourn (University of Edinburgh) - Health Data Scientist  Anne Richmond (University of Edinburgh) - Bioinformatics Analyst  Sarah Robertson (University of Edinburgh) - Young Person Engagement & Recruitment Co-ordinator  Rosie Tatham (University of Edinburgh) – Administrator  Alex Wood (University of Edinburgh) - Data Analyst / Developer  Dr Zhuoni Xiao (University of Edinburgh) – Research Fellow |
