# Supplementary material for: Effectiveness and Costs of Participant Recruitment Strategies to a Web-Based Population Cohort: Observational Study
Source: J Med Internet Res. 2025 Oct 6;27:e75116. doi: 10.2196/75116 (PMC12500226; doi:10.2196/75116)
Supplement: Multimedia Appendix 2 [file jmir-v27-e75116-s002.docx]

## Multimedia Appendix 2

**Table S1.** Chi-square test results comparing the proportion of individuals who returned their saliva sample kit or completed at least one follow up survey by recruitment method and demographic factors.

|  | **N** | **df** | **X^2^** | **p value** |
| --- | --- | --- | --- | --- |
| **Saliva sample return** | | | | |
| Recruitment method | 7836 | 5 | 148.83 | 2.20E-16 |
| Sex | 7826 | 1 | 65.03 | 7.38E-16 |
| Age | 7836 | 2 | 287.06 | 2.20E-16 |
| Ethnicity | 7823 | 1 | 0.89 | 0.347 |
| SIMD | 7798 | 4 | 151.96 | 2.20E-16 |

**Table S2.** Pairwise comparisons of the proportion of saliva samples returned by recruitment method using Bonferroni correction. Table of p value results.

|  | **COVIDLife Invitation** | **News Media** | **Snowball** | **Social Media** | **TV** |
| --- | --- | --- | --- | --- | --- |
| **News Media** | 1 |  |  |  |  |
| **Snowball** | 3.24E-07 | 0.002241 |  |  |  |
| **Social Media** | 8.88E-22 | 8.7E-09 | 0.471226 |  |  |
| **TV** | 1.31E-20 | 2.93E-09 | 0.156751 | 1 |  |
| Assorted methods/Unknown | 4.16E-10 | 6.7E-06 | 0.949044 | 1 | 1 |

**Table S3.** Pairwise comparisons of the proportion of saliva samples returned by age group using Bonferroni correction. Table of p value results.

|  | **<35 years** | **>60 years** |
| --- | --- | --- |
| **>60 years** | 1.5E-43 |  |
| **35 to 60 years** | 0.000707 | 6.08E-49 |

**Table S4.** Pairwise comparisons of the proportion of saliva samples returned by SIMD using Bonferroni correction. Table of p value results.

|  | **1 - Most Deprived** | **2** | **3** | **4** |
| --- | --- | --- | --- | --- |
| **2** | 0.007969 |  |  |  |
| **3** | 6.39E-09 | 0.044431 |  |  |
| **4** | 5.41E-16 | 4.23E-06 | 0.331909 |  |
| **5 - Least Deprived** | 2.54E-28 | 9.83E-15 | 2.42E-06 | 0.020081 |
